# Neurocognitive Impairment After Propofol With Relevance for Neurosurgical Patients and Awake Craniotomies—A Prospective Observational Study

**DOI:** 10.3389/fphar.2021.632887

**Published:** 2021-02-18

**Authors:** Nina Zech, Milena Seemann, Ralf Luerding, Christian Doenitz, Florian Zeman, Hamit Cananoglu, Martin G. Kees, Ernil Hansen

**Affiliations:** ^1^ Department of Anesthesiology, University Hospital Regensburg, Regensburg, Germany; ^2^ Department of Neurology, University Hospital Regensburg, Regensburg, Germany; ^3^ Department of Neurosurgery, University Hospital Regensburg, Regensburg, Germany; ^4^ Centre for Clinical Studies, University Hospital Regensburg, Regensburg, Germany

**Keywords:** propofol, awake craniotomy, neurocognitive impairment, digit span, trail making, word fluency

## Abstract

**Background:** Short-acting anesthetics are used for rapid recovery, especially for neurological testing during awake craniotomy. Extent and duration of neurocognitive impairment are ambiguous.

**Methods:** Prospective evaluation of patients undergoing craniotomy for tumor resection during general anesthesia with propofol (N of craniotomies = 35). Lexical word fluency, digit span and trail making were tested preoperatively and up to 24 h after extubation. Results were stratified for age, tumor localization and hemisphere of surgery. Results in digit span test were compared to 21 patients during awake craniotomies.

**Results:** Word fluency was reduced to 30, 33, 47, and 87% of preoperative values 10, 30, 60 min and 24 h after extubation, respectively. Digit span was decreased to 41, 47, 55, and 86%. Performances were still significantly impaired 24 h after extubation, especially in elderly. Results of digit span test were not worse in patients with left hemisphere surgery. Significance of difference to baseline remained, when patients with left or frontal lesions, i.e., brain areas essential for these tests, were excluded from analysis. Time for trail making was increased by 87% at 1 h after extubation, and recovered within 24 h. In 21 patients undergoing awake craniotomies without pharmacological sedation, digit span was unaffected during intraoperative testing.

**Conclusion:** Selected aspects of higher cognitive functions are compromised for up to 24 h after propofol anesthesia for craniotomy. Propofol and the direct effects of surgical resection on brain networks may be two major factors contributing (possibly jointly) to the observed deficits. Neurocognitive testing was unimpaired in patients undergoing awake craniotomies without sedation.

## Introduction

Intraoperative neurological monitoring and testing is indicated for neurosurgical interventions like deep brain stimulation (DBS) and resection of tumors in eloquent brain areas. For these procedures “awake craniotomy” is the favored approach, usually in an “asleep-awake-asleep” (AAA) technique or in “monitored anesthesia care” (MAC), where anesthesia or sedation with or without airway management is intermittently interrupted for testing (Heo et al., 2008; Bilotta and Rosa, 2009; Paldor et al., 2016). However, the applied anesthetics can interfere with intraoperative hemodynamic stability, airway safety, respiration and cooperation, and orientation, or even mimic neurological deficits (Venkatraghavan et al., 2006).

During surgery for low grade glioma in the vicinity of motoric or language brain areas anesthetics can interfere with test sensitivity in brain mapping. Small functional impairments like slurred or delayed speech have significant consequences, and differentiation of its origin, surgery or anesthetics, is essential (Ott et al., 2014). Similarly, for optimizing DBS outcomes, the functional accuracy as determined by intraoperative electrophysiological data as well as test stimulations to assess adverse effects is critical (Venkatraghavan et al., 2006). Therefore, the optimal management of anesthesia for awake craniotomies and especially the depth of sedation reaching from conscious sedation to general anesthesia are still under debate (Arzoine et al., 2020; Madadaki et al., 2020). We recently have described an “awake-awake-awake-technique” without any sedation, using cranial nerve blocks for analgesia and therapeutic communication to guide the patients (Hansen et al., 2013; Lange et al., 2015).

In clinical practice one of the most often used sedatives is propofol due to its short action and well controllability. Considering that testing is intended within minutes after stopping drug infusion, the meaning of “short acting drug” needs discussion, as well as its impact on higher, more complex neurocognitive brain function than postoperative alertness. Any statement about neurocognitive impairment and recovery after application of sedatives such as propofol is highly dependent on the test used. Evaluation of simple reactions and defensive reflexes after general anesthesia show recovery from propofol after minutes; evaluation of recognition and memory reveals impairment for hours; much longer cognitive impairment for days and weeks has been described as postoperative cognitive deficit (POCD) with specific test batteries. In neurosurgical patients with brain tumors and especially with tumors in eloquent areas of the brain operated in awake craniotomy, specific neurocognitive tests are applied (Lezak et al., 2012; Rofes et al., 2017). In a previous study we have demonstrated impaired cognitive and motor function in non-neurosurgical patients after surgery in local anesthesia with slight sedation (RAS) or in total intravenous anesthesia (TIVA) (Ott et al., 2014). These findings assessed in ENT- or orthopedic patients, could be even more relevant in neurosurgical patients, where such data are limited. Therefore, the aim of the present prospective clinical study was to evaluate the time-dependent effect of propofol in neurosurgical patients and on a neuropsychological test battery commonly used in neurological and neurosurgical patients, namely word fluency, digit span and trail making.

## Methods

34 patients undergoing 35 craniotomies for tumor resection were enrolled in this prospective observational study (one patient was operated twice because of local tumor recurrence). Ethical approval of the study (no. 12–101–0007) was provided by the ethics committee of the University Hospital Regensburg, Regensburg, Germany (Chairperson: Prof. Dr Ch. Stroszczynski) on January 24, 2012. Written informed consent was obtained from all patients. Exclusion criteria were: age under 18 years, severe systemic disease presenting a constant threat to life (ASA IV), language barriers, severe psychiatric disease, neurological disorders or pre-existing cognitive impairments.

All patients received a total intravenous anesthesia under standardized conditions, without any premedication. Anesthesia was induced with fentanyl (0.2–0.3 mg), propofol (2–3 mg kg^−1^ body weight) and rocuronium (0.5–0.6 mg kg^−1^ body weight). Anesthesia was maintained starting with a continuous infusion of propofol (6–10 mg kg body weight^−1^ h^−1^) and remifentanil (0.3–0.5 μg kg body weight^−1^ min^−1^). Depth of anesthesia was monitored by bispectral index (BIS Vista™, Covidien) and propofol dose adjusted to a strict target BIS of 40 ± 5 (BIS-controlled TIVA). A continuous infusion of norepinephrine was titrated if necessary, to avoid a mean arterial pressure below 65 mmHg. 8 mg of dexamethasone was given to avoid intraoperative brain swelling and oedema. At the end of surgery, patients received parecoxib (40 mg) or metamizol (1.25 g) for adequate postoperative analgesia.

At the end of surgery, all patients remained intubated for transport to the ICU. After stable haemodynamic and respiratory conditions, the continuous infusion of propofol and remifentanil was stopped. Criteria for extubation were sufficient breathing, recovered defensive reflexes, and ability to follow simple instructions, e.g., squeezing of the hand.

### Neurological Function Tests

To examine the patients` general alertness, ability to concentrate and higher cognitive functions, they performed three neurological function tests. Time dependent recovery was evaluated, as patients performed each test several times starting one day before surgery for a preoperative baseline prior to any sedation. The tests were repeated 10 min, 30 min and 60 min after extubation as well as after 24 h.

### Word Fluency

To assess the patients´ lexical verbal fluency, the Regensburg Word Fluency Test (RWT), the German version of the Controlled Oral Word Association Test was conducted, a test for divergent thinking solving (Lezak et al., 2012). The patients were asked to name as many words with a given initial letter as possible within 1 min. Letters were “B” preoperatively, and “K”, “M” or “S” at the other times of testing, respectively.

### Digit Span

Verbal working-memory as part of the short-term memory, and the patients´ attention was measured with the digit span forward and backward from the German version of the Wechsler Adult Intelligence Scale (WAIS-R), hereinafter referred to as Digit Span Test (DST) (Lezak et al., 2012). First, the examiner reads aloud a series of three digits to the patient, who has to repeat it. The procedure is repeated with a series extended by one digit and so on. If the patient fails in two consecutive series of equal length, the test is stopped. In the second part the patient has to repeat the series of digits in reverse order. The scores of the first and second part are summed up.

### Trail Making

The Trail Making Test (TMT), a part of the Halstead-Reitan-Test, is providing a measure of complex visuoperceptual tracking, planning and flexibility (Lezak et al., 2012). Only subtest A was used. Here, the patients’ velocity of information processing is assessed by having to connect numbers from 1 to 25 in the right order, which are randomly spread over a sheet of paper (size DIN A4, 210 × 297 mm). The time to connect all numbers with immediate correction of any mistakes is measured. Testing 10 and 30 min after extubation was omitted, because patients regularly were too tired to fulfill this sophisticated task at this time.

### Awake Craniotomies

Test results for DST were compared to those from 21 patients undergoing awake craniotomies for tumor resections in the awake-awake-awake-technique (Hansen et al., 2013). There, DST was tested one week preoperatively, as well as during surgery, i.e., immediately after craniotomy and prior to brain mapping. Depending on tumor localization and pre-existing deficits, patients performed further neurocognitive or motor function tests intraoperatively (data not shown).

### Statistical Analyses

For clinical assessment, raw scores were transformed into z-scores adjusting for age, sex and education, using population-based normative data provided by the respective test author. A z-score of -1, i.e., an individual’s performance is below 1 standard deviation (SD) of the average of normal, healthy, age- and education-matched controls, was chosen as cut-off value to indicate impaired performance.

Statistical analyses were performed with SPSS 25.0. Variables were tested for normality using the Kolmogorov-Smirnov-Lilliefors Test. Parametric data are presented as means and SD, and were compared between groups by using an Analysis of Variance with posthoc pairwise comparison. Non-parametric data (TMT results) are presented as median and interquartile range (IQR), and groups were compared using Wilcoxon rank-sum-test. Relations between variables were shown with Pearson’s correlation coefficient (r). Statistical significance was accepted at a *p* ≤ 0.05.

## Results

### Patients Characteristics

Thirty-four patients (22 females, 12 males) undergoing 35 procedures fulfilled the inclusion criteria and were enrolled in the study. Age ranged from 22 to 81 years with a median of 60 years. Operating time (incision to suture) was between 116–192 min, with an anesthesia time (induction to cessation of propofol, including transport to ICU) of 349.5 ± 88.2 min. Cumulative propofol dose was 2,395 ± 697 mg, with a propofol rate of 6.1 ± 0.6 mg kg^−1^ h^−1^. Tumors were located in the left hemisphere in nine patients, in the frontal lobe in eight patients. Time from stop of propofol to extubation was 35.2 ± 18.5 min.

### Word Fluency

**FIGURE 1:**
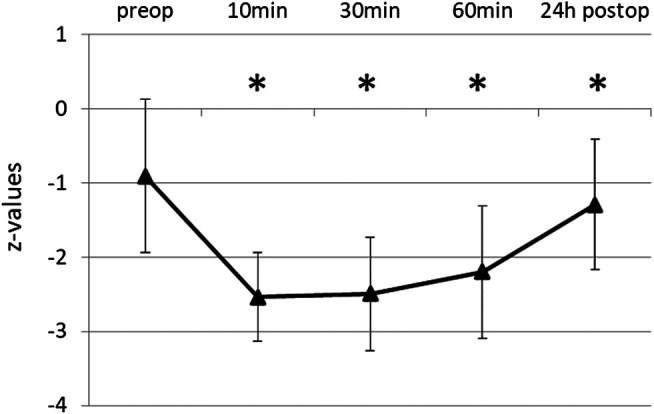
Word Fluency Test (RWT) before and after propofol. Lexical word fluency was tested by RWT preoperatively and at various times after extubation. Means and SD of z-values (normalization for age and gender) are given. N = 35, *p ≤ 0.05 compared to preop.

The initial score of RWT was 9.26 ± 3.37, the corresponding z-value -0.91 ± 1.03, i.e., near the lower border of the normal range. Word fluency was significantly impaired after propofol (Figure 1). Average test score was reduced to 30% of the baseline 10 min after extubation, and for 1 h remained below 50%. After 24 h word fluency had recovered to 87% (8.06 ± 3.75, z = -1.29 ± 0.88), but still significantly different to baseline (*p* = 0.009). Percentages refer to the baseline of original score values.

### Digit Span

**FIGURE 2:**
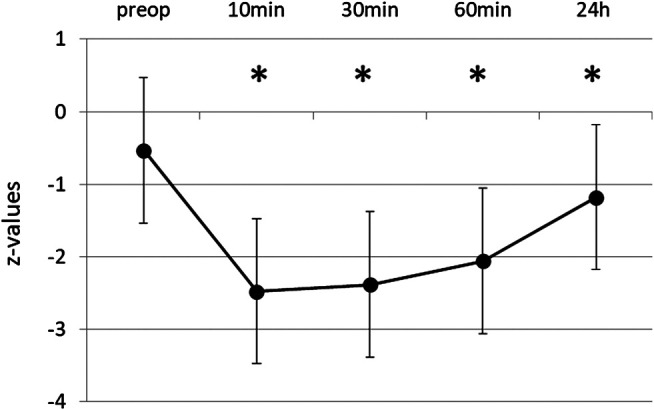
Digit Span Test before and after propofol. Repeating series of digits forwards and backwards was tested preoperatively and at various times after extubation. Means and SD of z-values (normalization for age and gender) are given. N = 35, *p ≤ 0.05 compared to preop.

**FIGURE 3:**
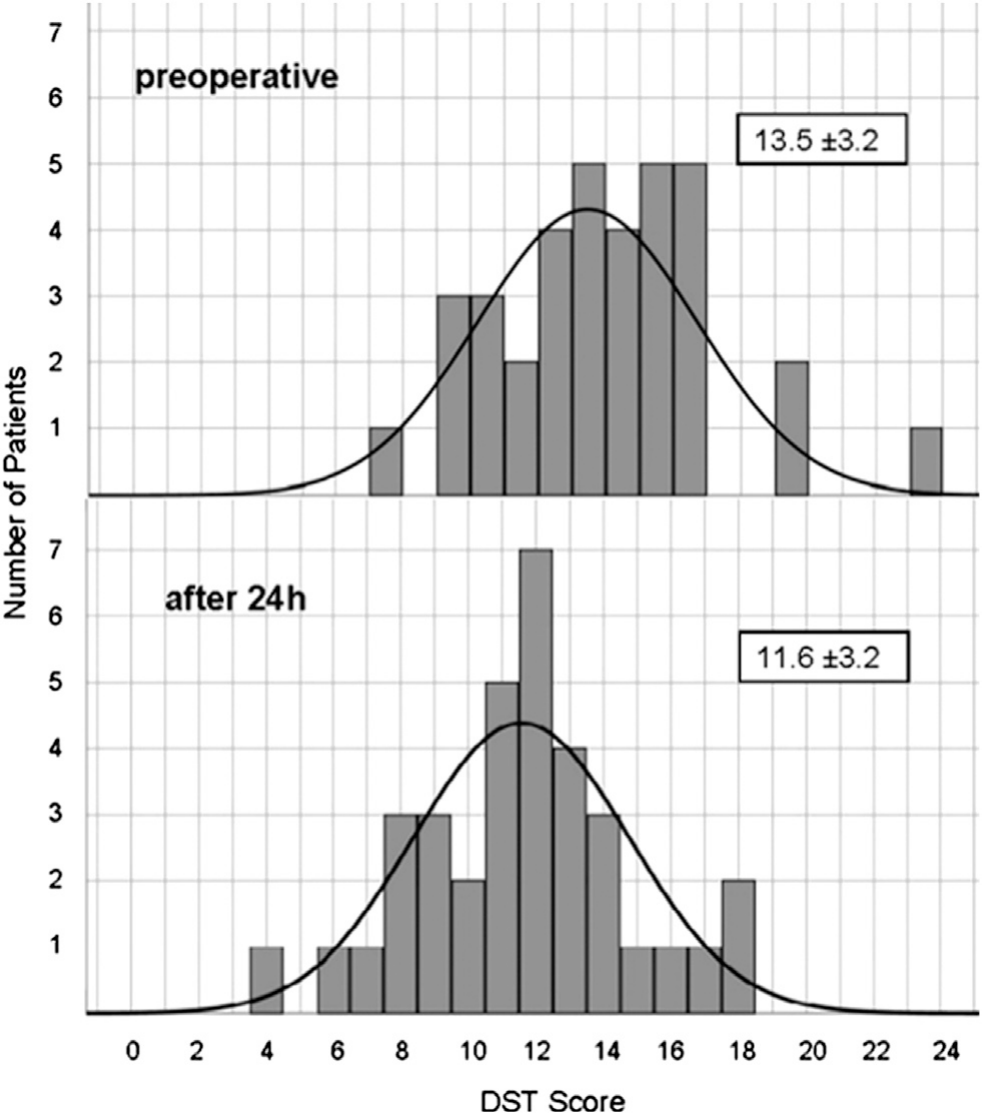
Distribution of Digit Span Test score before and 24 h after propofol. Number of patients reaching a certain DST score before and 24 h after anesthesia.

The initial mean score in DST was 13.46 ± 3.24 and the corresponding z-value within the normal range (-0.53 ± 0.93). Performance was significantly impaired after propofol (Figure 2). 10 min after extubation the test score was reduced on average to 41% of the initial score value. For up to 1 h, performance still did not exceed 55% of original score. After 24 h digit span had recovered to 86% (11.57 ± 3.18, z = -1.12 ± 0.97), still significantly different to baseline (*p* ≤ 0.001). Figure 3 shows the distribution of baseline and 24 h values.

### Trail Making

**FIGURE 4:**
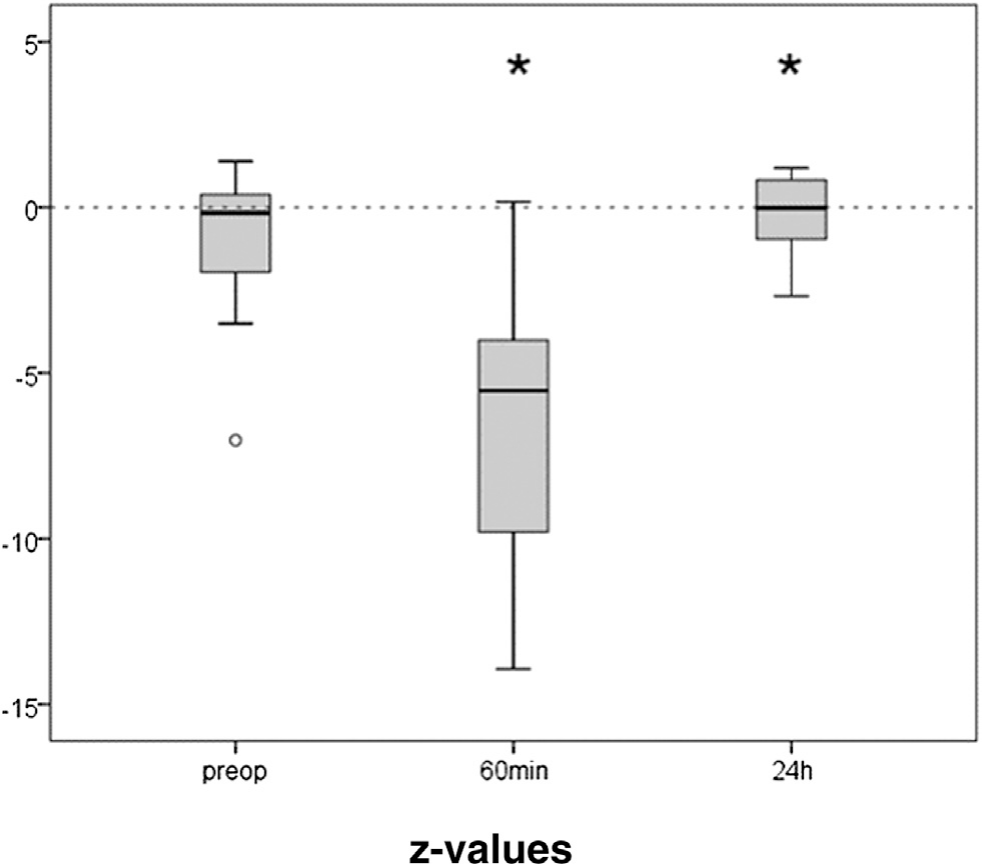
Trail Making Test before and after propofol. The capability to connect numbers from 1 to 25 randomly spread over a sheet of paper in the right order, was tested preoperatively, 1 h and 24 h after extubation. Medians and interquartile ranges of z-values (normalization for age and gender) are given, after elimination of patients not able to perform the test after 1 h. N = 21, *p ≤ 0.05 compared to preop.

The preoperative results of the TMT averaged 47 (IQR 38) seconds. The initial z-value derived thereof was median −0.88 (IQR 2.99), i.e., at the low normal range. Only the 21 patients that were able to perform the test 60 min after extubation were included in the further analysis. They showed an initial median z-value of −0.17 (IQR 2.51). Trail making was significantly impaired after anesthesia (Figure 4). One hour after extubation the time needed to perform the task was more than doubled (88s, z = −5.54, IQR 6.57; *p* ≤ 0.001). After 24 h performance was completely recovered (z = −0.02, IQR = 1.92; *p* = 0.028).

### Contributing Factors

Comparing the preoperative and the 24 h-postoperative values no differences between male and female patients were observed. Age had an impact on the results. 24 h after extubation the performance in RWT and DST was still impaired (z -score < −1) in both younger (<60 years) and older (>60 years) patients (Table 1). However, the difference to baseline was significant only in the elderly, since patients of the younger cohort started out at a lower level. Also, side and localization of tumor and surgery had effects on performance and recovery (Table 1). There was no significant correlation between cumulative propofol dose and difference between pre- and 24 h postoperative DST z-values (r = 0.26, *p* = 0.14) and for RWT z-values (r = 0.05, *p* = 0.82), respectively. Similarly, no significant correlation was found between anesthesia time and differences between pre- and 24 h postoperative z-values for DST (r = −0.01, *p* = 0.97) and RWT (r = 0.03, *p* = 0.89), respectively. Linear regression analyses showed no correlation between cumulative or body weight adjusted propofol dose, respectively, and test performance at any time point.

**TABLE 1:** Impact of age and localization of tumor and surgery on RWT and DST. Means of values normalized for age and gender (z-values) are given.

	RWT			DST		
	Pre	24 h	p*	Pre	24 h	p*
All (N = 35)	−0.91	−1.29	0.009	−0.53	−1.12	0.004
<60 years (N = 19)	−1.10	−1.40	0.265	−0.67	−1.00	0.460
≥60 years (N = 16)	−0.35	−1.15	0.009	−0.33	−0.99	0.010
p**	0.043	0.379		0.503	0.821	
left (N = 9)	−1.444	−2.078	0.100	−0.591	−0.852	0.609
without Left (N = 26)	−0.723	−1.023	0.053	−0.513	−1.218	0.001
p**	0.070	0.001		0.832	0.336	
frontal (N = 8)	−0.638	−1.338	0.097	−0.749	−1.210	0.414
without Frontal (N = 27)	−0.989	−1.282	0.054	−0.469	−1.098	0.004
p**	0.406	0.877		0.464	0.779	

RWT = lexical Word Fluency Test, DST = Digit Span Test. left = patients with tumor and surgery in left hemisphere, "without left” = all patients except those with left side lesion (tumor and surgery), “without frontal” = all patients except with frontal lesion (tumor and/or surgery). Statistical analysis of independent (p**) and dependent (p*) variables by student-T-test.

### Awake Craniotomies

**FIGURE 5:**
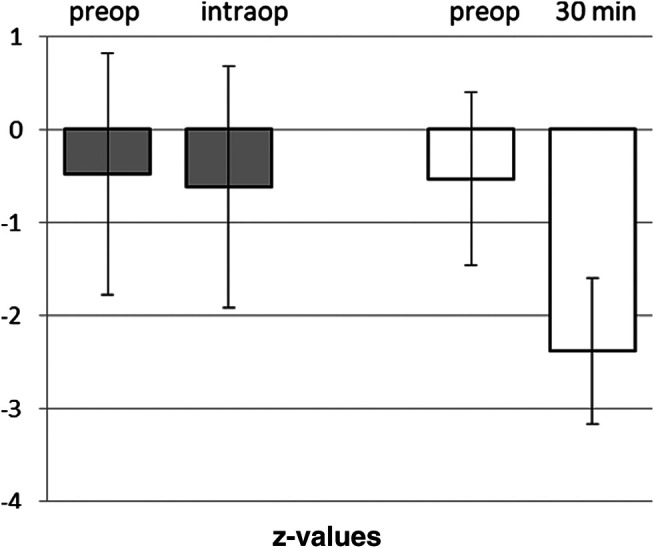
Comparison of Digital Span Test in awake craniotomies vs. craniotomies under total intravenous anesthesia. Awake craniotomies in the awake-awake-awake-technique (without sedation), pre- and intra-operatively (*n* = 21). Propofol-based anesthesia for craniotomies, pre- and post-operatively (30 min). Means and SD of z-vaues (normalization for age and gender) are given.

The twenty-one patients undergoing an awake craniotomy in the awake-awake-awake-technique for resection of a tumor in eloquent brain area had a baseline in DST of z = −0.48 ± 1.35. Intraoperatively, at the beginning of the test phase with brain mapping, the patients showed a mean z-value of −0.62 ± 1.35, not significantly different from baseline (*p* = 0.739) (Figure 5). In 11 out of the 21 patients the intraoperative scores were unchanged or even better than the preoperative values.

## Discussion

Pharmacokinetic and pharmacodynamic data cannot directly be applied to neurosurgical patients and the effect of propofol on an injured or operated brain, as well as the combined effect of propofol and surgery, are still unclear. In consequence, although a patient is assessed awake with regard to gross verbal responses, residual anesthetic effects may persist and impair neurological assessment or speech testing for brain mapping and resection extent during awake craniotomy.

### Deep Impairment and Slow Recovery

Considerable and significant impairment of word fluency, digit span and trail making was demonstrated for at least 1 h. All three neurocognitive tests commonly used for neurological examinations in neurosurgery are well established in epilepsy surgery programs, in which they were validated in lesion studies and in PET-activation studies (Jokeit et al., 1997). Hereby, calculation of z-values, i.e., data normalized for age and gender, allows for much more precise comparison than using rather small control groups due to large population based normative data. The results recovered only to −2.2 or −2.0 for RWT or DST, respectively. Thus, the performance that preoperatively was decreased but still within the normal range in these neurosurgical patients, remained more than two standard deviations below normal values for at least 1 h after extubation. Depression in the TMT was even more pronounced with a more than doubled time to perform the test and a z-value of −5.5 1 h after extubation. These documented impairments are well within the period relevant for neurological testing during awake craniotomy or after neurosurgery. We observed a significant impairment in RWT and DST for 24 h. Therefore, a fast recovery of consciousness, vital functions and reflexes is contrasted by a slow recovery of higher mental functions. After 24 h the scores of DST (see Figure 3) and RWT (data not shown) still were normally distributed as evidence for the reaction of all participant, whereas a strong reaction of a few patients as explanation would result in skewness of the distribution or in a second peak. Others have found temporary neurocognitive deficits with other tests. Sanou et al. observed reduced performance in five cognitive tasks covering memory and language comprehension for up to 3 h after a short-term sedation for coloscopy with propofol, but complete recovery after 6 h (Sanou et al., 1996). Beyond the tested period, much longer cognitive impairment after surgery and anesthesia has been described as postoperative cognitive deficits (POCD) (Höcker et al., 2009; Royse et al., 2011; Silbert et al., 2014). With much longer observational periods, complex test batteries and various discussed causes, our results should not be mixed up with POCD.

### Correlation to Pharmacokinetics

From a pharmacokinetic point of view, propofol is a well controllable hypnotic agent, with rapid onset and elimination, huge volume of distribution and high clearance (Larsen et al., 2000; Schüttler and Ihmen, 2000; Sahinovic et al., 2018). The most accepted pharmacokinetic models are linear with three compartments. Accordingly, elimination of propofol is described with an elimination clearance (Cl1) and the inter-compartmental clearance (Cl2 and Cl3), which are partly influenced by age, weight and gender and three different half times (T1/2 α, β and γ) (Schüttler and Ihmen, 2000). It has a comparatively high elimination clearance, which even exceeds hepatic blood flow (Hiraoka et al., 2005). Nonetheless, it accumulates due to extensive distribution into peripheral compartments and has a long terminal half-life of about 6 h. Gepts and colleagues determined blood concentrations during and after propofol infusions for 2–4 h at 3, six or 9 mg/kg/h; after stopping the infusion, 2–3 h elapsed before blood concentrations declined to <10% of steady state concentrations (Gepts et al., 1987). It is likely that the long terminal half-life and slow elimination is constrained by a slow back-diffusion from the deep tissue compartment. In contrast, the rapid recovery, i.e., the decline in propofol blood concentration to a sub-anesthetic level is dominated also by distribution (Schüttler and Ihmen, 2000). Prolonged effects on higher cognitive functions are therefore plausible. Only few studies have measured pharmacokinetics of propofol in patients with brain tumors (Sahinovic et al., 2014; Sahinovic et al., 2017), or during awake craniotomy (Lobo and Beiras, 2007), with varying results.

### Impairment of Neurocognitive Tests by Anesthetics

Numerous studies have reported on impairment of the central nervous system after anesthesia or sedation using propofol (Heath et al., 1990; Bilotta et al., 2007; Höcker et al., 2009; Royse et al., 2011; Shen et al., 2013). The level and duration of reduced performance are varying widely with the applied tests. Tiredness, drowsiness and impaired alertness were observed for 1 h, and decreased performance in the William’s memory function test, i. e., the recall of pictures, for 2 h. The authors state a deficit lasting 24 h in the Wechsler logical function passage testing recall of details from a short story, however only in comparison to a control group consisting of nurses, not when compared to the preoperative value (Heath et al., 1990). Others have observed delayed recovery of learning and memory, specifically the storage of new verbal information; after a 30 min propofol anesthesia (N’Kaoua et al., 2002). However, impairment for 24 h was only detectable for deep level semantic processing, not for shallow sensory, phonetic processing, and only for cued recall, not for free recall. On the other hand, this study provides evidence for long term propofol effects even after short-time application. Other neurocognitive tests such as recognition and memory tasks including implicit perceptional facilitation were found not to be affected by propofol (Polster et al., 1993).

Only few studies are available regarding the consequences of propofol pharmacokinetics in neurosurgical patients. In a study on early postoperative cognitive recovery after TIVA for supratentorial craniotomy for instance reduced performance was found after 45 min and complete recovery after 3 h in two test batteries (Bilotta et al., 2007). Only rarely have the compromising effects of propofol been evaluated with neurocognitive tests used in neurosurgical patients and for awake craniotomy. Impairment in DST was observed 1 h after anesthesia with induction by propofol, but not after 3, or 24 h, respectively (Sanders et al., 1989). After TIVA with propofol for interventional embolization of cerebral aneurysm, DST was found impaired for 1 h. A controlled word association test was significant lower for up to 2 h (Münte et al., 2001).

In the present study, the results of our prior trial evaluating DST and RWT in non-neurosurgical patients were confirmed in patients undergoing craniotomy, and extended to an observation period of 24 h (Ott et al., 2014). The lower starting level, the deeper and longer impairment reflects the situation of brain disease and surgery in which propofol displays its effects relevant for neurological testing in these patients.

### Limitations

Beyond propofol, opioid effects, motivational aspects, effects of vigilance and arousal, and finally effects of the brain tumor itself and the surgery (like deformations around the cavity, changes in the cerebrospinal liquor flow and pneumocephalus) have to be considered in the neurosurgical patients under examination, i.e., pre-existing and post-operative neurological deficits. For scientific reasons it may seem preferable to conduct a randomized trial or to study minor neurosurgical procedures to separate tumor and surgery effects from anesthetic effects. However, the complex situation studied here is of clinical interest and relevance, in particular for awake craniotomies. The influence of tumor, surgery and propofol on postoperative neurocognitive performance, including the effects of anesthesics on the injured brain, is complex, but the clinical reality. Nevertheless, many of impairments by tumor and operation do not cease within 24 h as observed here. Our patients had not received sedatives other than propofol, especially no benzodiazepines. However, analgesics applied with the propofol might have added to the observed effects. No effects of opioids on neurocognitive tests like DST have been observed in chronic opioid medication (Kurita and de Mattos Pimenta, 2008; Panjabi et al., 2008; Diasso et al., 2019). For instance, in a study on patients with Parkinson’s disease no decrement in cognitive neuropsychological performances was found after pain therapy with tapentadol (Freo et al., 2018). But also studies on shorter acting opioids like fentanyl or alfentanil show no effect on learning and recall, or memory of words and pictures, both after low or high doses (Scamman et al., 1984; Veselis et al., 1997). Pain is known to impair neurocognitive performance, while adequate analgesia prevents reduction and even improvement can be observed (Freo et al., 2018). Therefore, proper pain management is essential when using these tests. In the patients for awake-craniotomy following our protocol without sedation and varying doses of remifentanil, we did not observe differences in vigilance during DST and neurological testing, whether they had received opioids or not.

Especially the observed prolonged effect on these neurocognitive tests, relevant and commonly used in patients with brain tumors, even after 24 h is a novel result and worth to discuss. It is reasonable to suspect that the surgery of the brain might be responsible rather than the anesthesia. In that case however, the impairment should be more pronounced in left hemisphere and frontal lesions, since the left middle frontal lobe is essential for performance in RWT, and the left dorsolateral prefrontal cortex for DST (Reuter-Lorenz et al., 2000; Senhorini et al., 2011). This was not the case (Table 1). In DST, values of patients with left-sided or frontal lesions were not significantly different to the rest of the patients. In RWT the lower performance of left-side-operated patients can be explained by their lower baseline. Moreover, the discrepancy of 24 h and preoperative values of both RWT and DST remained significant after exclusion of patients operated on the left hemisphere or on the frontal lobe (“without left” and “without frontal” in Table 1). Instead, in patients with left-sided or frontal resection the difference to baseline was not significant, i.e., the effect of localization is no larger than the propofol effect. This makes it unlikely that surgical trauma of brain areas relevant in these tests, (e.g. the left hemisphere for DST and RWT) was the cause of reduced performance. For more clarity, further studies comparing different anesthetic regimes for non-neurosurgical operations, standard procedures and awake craniotomies for cerebral tumor resection in different localizations with conduction of the same neurocognitive tests at comparable time points are needed.

### Impact for Awake Craniotomies

Our intention was to relate our results to the situation of the testing phase of awake craniotomy, although patients not of the same study. In awake craniotomies it is critical to start with brain mapping and neurological tests as soon as possible after cessation of propofol or extubation. After BIS-guided propofol anesthesia Soehle et al. measured return of consciousness at BIS of 77 and propofol plasma concentration of 1.2 µg/mL. Neurological testing was possible as soon as the BIS had increased to 92 ± 6 and measured plasma concentration had decreased to 0.8 µµg/ml. This translated to a time delay of 23 ± 12 min between return of consciousness and begin of neurological testing (Soehle et al., 2018). Our data indicate residual neurocognitive impairment at the time of intraoperative tests. Notably, both DST and RWT are relevant to verbal competence in patients with surgery in eloquent brain areas. Of course, the question is justified whether results of a propofol application for 5–6 h are relevant for a clinical situation where it is applied for 1–2 h like in awake craniotomy prior to the awake test phase, or a dosage of 3–12 mg kg^−1^ h^−1^ as used in TIVA or AAA comparable to one of 1–8 mg kg^−1^ h^−1^ used in MAC (Stevanovic et al., 2016). The mean dosage of 6.1 mg kg^−1^ h^−1^ in our study might be considered relevant to both situations. And the dependence of wake-up time and neurocognitive recovery after propofol on dosage or duration is not linear (Sahinovic et al., 2017), so that an effect demonstrated for 24 h might correlate to an impairment for at least 12 h in the awake craniotomy situation. In a study on propofol for conscious sedation (MAC) in awake craniotomy, for instance, a total propofol dose of 1900 mg was reported (Shen et al., 2013), not far different from the 2,400 mg in our study.

Various efforts have been made to reduce or avoid sedation prior to the neurological tests. In our experience, therapeutic communication and relationship can render sedation unnecessary (Hansen et al., 2013). Test results of our patients during awake craniotomies without sedation based on cranial nerve blocks and therapeutic communication prove unimpaired neurocognitive competence. The comparison to DST results during awake craniotomies is limited by the fact, that the two groups of patients were not part of one study and a comparable study procedure. Further studies are needed where the performance in these neurocognitive tests and other tests relevant for surgery in motoric or eloquent brain areas is evaluated after propofol application in the pre-test phase or without propofol by its avoidance facilitated by therapeutic communication or hypnosis. Moreover, studies should clarify, whether an observed higher neurocognitive competence in this operative phase can actually lead to more precise tumor resection with conserved ability of speech, and better neurological outcome. For the meantime our results add some information for the transfer of propofol pharmacokinetics to clinical neurosurgery by studying neurosurgical patients and by using tests commonly applied in these patients.

## Conclusion

Higher cognitive functions, namely word fluency and digit span are compromised for up to 24 h after propofol application for craniotomies. Neither the tumor localization nor the brain surgery could solely account for this prolonged impact. In contrast, during awake craniotomy in the awake-awake-awake-technique no worsening for DST could be found. Our results add evidence that efforts to minimize sedation in awake craniotomies are appropriate.

## Data Availability

The raw data supporting the conclusion of this article will be made available by the authors, without undue reservation.

## Abbreviations

: BIS, bispectral index; DBS, deep brain stimulation; DST, digit span test; ENT, ear nose and throat; ICU, intensive care unit; IQR, interquartile range; PCF, posterior cranial fossa; POCD, Postoperative cognitive deficit; RWT, Regensburg word fluency test; TMT, trail making test; WAIS-R, Wechsler Adult Intelligence Scale.
